# Distortions to the passage of time for annual events: Exploring why Christmas and Ramadan feel like they come around more quickly each year

**DOI:** 10.1371/journal.pone.0304660

**Published:** 2024-07-10

**Authors:** Ruth Ogden, Saad S. J. Alatrany, Ashraf Muwafaq Flaiyah, Hasan ALi Sayyid ALdrraji, Hanan Musa, Abbas S. S. Alatrany, Dhiya Al-Jumeily

**Affiliations:** 1 School of Psychology, Liverpool John Moores University, Liverpool, United Kingdom; 2 Imam Ja’afar Al-Sadiq University, Baghdad, Iraq; 3 Ibn Reshed College of Education for Human Sciences, University of Baghdad, Baghdad, Iraq; 4 Baghdad Centre for Psychosocial Support, Baghdad, Iraq; 5 University of Information Technology and Communications, Baghdad, Iraq; 6 School of Computer Science and Mathematics, Liverpool John Moores University, Liverpool, United Kingdom; 7 NIHR Leicester Biomedical Research Centre, University of Leicester, Leicester, United Kingdom; Emory University, School of Public Health, UNITED STATES

## Abstract

**Background:**

Commonly heard statements such as “*Christmas comes around more quickly each year*” suggest that the passage of time between annual events can become distorted, leading to the sensation of time passing more quickly than normal. At present however, it is unclear how prevalent such beliefs are and, what factors are predictive of it.

**Aim:**

To explore the prevalence of beliefs that annual events such as Christmas (Study 1 UK sample) and Ramadan (Study 2 Iraqi sample) feel like they come around more quickly each year. To establish the association between distortions to the passage of time between annual events and emotional wellbeing, event specific enjoyment, memory function and self-reported attention to time.

**Methods:**

Participants completed an online questionnaire exploring their subjective experience of time in relation to Christmas and Ramadan. In addition, measures of attention to time, memory function, quality of life and event specific emotion were also taken.

**Findings:**

There was widespread agreement that Christmas and Ramadan appeared to come around more quickly each year. In both countries, this belief was associated with greater prospective memory errors, greater attention to time and greater enjoyment of the event. Furthermore, in the UK greater belief that Christmas comes around more quickly was associated with lower social quality of life and in Iraq, greater belief that Ramadan comes around more quickly each year was associated with lower age and female gender.

**Conclusions:**

Distortions to the passage of time for annual events are widespread, occur across multiple cultures and are consistently predicted by prospective function, event enjoyment and attention to time. The absence of an association between older age (above 55 years) and a faster passage of time suggests that caution should be taken when concluding that time passes more quickly with increasing age.

## Introduction

Human experience of the passage of time is highly flexible and prone to distortions which can make time feel like time is passing more quickly or slowly than normal [1–19]. Distortions to time can occur over short epochs, for example, a meeting may feel like is flew or dragged depending on its content, however the widespread use of common adages such as “*Christmas comes around more quickly each year*” suggests distortions to time are also common for longer epochs. Despite the pervasiveness of these anecdotal reports, little is understood about why the passage of time seems to distort between annual events. The current study therefore sought to explore the prevalence and predictors of distortions to the passage of time for specific event which occur over long epochs. This will be achieved by examining beliefs that Christmas and Ramadan come around more quickly each year.

The idea that the speed of time changes over long epochs has previous been explored in studies of the effect of age on the passage of time. These studies suggest that whilst there is some support for the idea that time passes more quickly with age, the effect does not seem to be unique to the elderly, and instead appears to be present in adults of most ages [11–16]. For example, both Friedman & Janssens [12] and Wittmann et al’s., [11, 15] cross-sectional studies with 18–81 year olds exploring the extent to which age was a predictor of time experience concluded that, for the majority of measures of time experience (e.g. how quickly has today or this week felt like it was passing) age only accounted for a small proportion of the variance in experiences of time. The absence of age effects on experiences of time for short epochs (days and weeks) was also observed in cross-sectional studies conducted during the COVID-19 pandemic [1, 2], and in experience sampling method studies comparing older (69–78 years) and younger (20–23 years) [8].

Interestingly however, some studies which failed to find effects of age on time experience for short epoch, have noted them for longer ones, with participants perceptions of the speed of the past 10 years appearing to increase between the ages of 18 and 50 years [11–13, 15]. Studies of time experience in the very elderly (75 years and over), do however show age effects, however rather than time appearing to pass more quickly for this age group than for younger adults, time actually appears to pass more slowly [17].

Inconsistent effects of age on the perception of time may indicate that the other factors are primary determents of the speed of the passage of time. Many variables are known to influence experiences of the speed of the passage of time, for example, emotional experience, memory formation and attentional focus. It is feasible that individual differences in these factors contribute to the sensation that annual events such as Christmas and Ramadan come around more quickly each year.

### Emotional wellbeing

Christmas and Ramadan are emotion evoking events and the valence of the emotion that they evoke may distort passage of time judgments in relation to the events. Self-reported emotional states are consistently found to be associated with the subjective speed of the passage of time [1–11]. During COVID-19, a number of studies demonstrated that in the general population, positive mood is typically associated with the sensation that time is passing more quickly than normal whereas negative affect is associated with the sensation of time passing more slowly than normal [1–11, 18, 19]. These findings replicate those observed in pre-COVID-19 studies of distortions to the passage of time [18, 19] and are mirrored in clinical groups where time passing slowly is associated with increased depression [20–22], and lower wellbeing and quality of life [1–10, 23–25].

Emergent recent evidence from the COVID-19 pandemic also suggests that emotion impacts experiences of longer epochs such as years. Ogden & Piovesan (2022) [24] explored factors affecting distortions to peoples memory for the duration of the first 12-months of the COVID-19 pandemic. They observed that many people reported that the first-12 months felt longer than 12-months. This lengthening effect was associated with greater levels of depression, anxiety and social isolation. Individual differences in emotional appraisals of specific events, as well as broader long-term social circumstances such as isolation, poor psychological health and reduced wellbeing, therefore have the capacity to influence passage of time judgements for annual events.

Whilst the existing literature examining the impact of emotional states on the passage of time for epochs ranging from days to years suggests that changes in emotion can significantly distort the passage of time, existing studies have not specifically examined how the emotional appraisal of past and forthcoming events impact the passage of time for longer durations. It is therefore unclear whether the emotional appraisal of annual events such as Christmas and Ramadan would impact the passage of time in the same manner that emotional states such as depression, anxiety and stress do. The current study will therefore expand understanding of the role of emotional appraisal in passage of time judgements.

### Memory formation

Subjective changes in the passage of time may also be influenced by an individuals memory function [26–31]. How memory function affects time processing depends on the nature of the duration being judged. When time is being prospectively judged, and the individual is consciously aware that they are should be monitoring time throughout the event increased memory load and information processing load are associated with short perceptions of duration. However, when time is being judged in retrospect, and was thus not consciously monitored throughout the event, the effect of memory load on time experience reverses [26–31].

In general, for retrospective judgements of time, periods of time from which we can recall many distinct memories are perceived as having lasted for longer than those from which we can recall few memories [26–31]. Perceived time is therefore thought to be a function of memory storage size with greater memory storage associated with longer perceptions of time. The content of the memories recalled can also influence the perceived length of the event [28]. Memories which contain a high degree of contextual change or emotional change are generally associated with longer perceived lengths than those with little contextual or emotional change [29].

The importance of memory formation in time experience raises the possibility that fidelity of memory function may be an important factor in the experience of distortions to the passage of time, particularly across longer-epochs such as years. If memory impairment reduces the number of memories formed or recalled across a year, this may contribute to the sensation that the year has passed quickly. Indeed, it is conceivable that impaired memory function may contribute to experiences of distortions to the passage of time across the years.

### Attentional focus

How much attention is paid to time has a significant impact on the subjective speed of the passage of time. In the moment, the amount of attention paid to time is determined by the cognitive resources available to process time [29–34]. In periods of low cognitive low, for example, there time spare cognitive resources are used to process time, and this results in time passing more slowly than normal. Conversely, when cognitive load is high and tasks are engrossing little attention is paid to time and it passes quickly [29–34].

The presence of a deadline alters the relationship between attention and the passage of time. When completing time sensitive tasks or when a deadline is looming and we do not feel like we have “enough time” we experience time pressure. Increased awareness of time resulting from time pressure produces the sensation of time passing quickly [11, 35–39]. Annual events such as Christmas and Ramadan are themselves deadlines and may therefore create heightened temporal awareness as people prepare for the event. It is therefore feasible that temporal awareness in the run up to these events may be predictive of experiences of the passage of time.

### The current study

Whilst there is good evidence that factors such as emotions, memory function and attention influence time experience over short epochs, few studies have explored how factors such as these may collectively influence experiences of the passage of time between specific events which occur on annual basis, namely subjective experiences of the passage of time between Christmas and Ramadan. In doing so, the study will also illuminate the factors which influence experiences of the passage of time over longer periods of time such as year-to-year. The current study therefore aimed to explore whether emotional wellbeing, event specific enjoyment, memory function and self-reported attention to time were related the sensation of annual events coming around more quickly each year. Experiences of time were assessed using passage of time judgment (POTJ) questions. In Study 1, conducted in the UK, the passage of time was assessed using an online questionnaire examining the extent to which participants agreed with the statement that “*Christmas comes around more quickly each year”*. In Study 2, conducted in Iraq, the passage of time was assessed using a further online questionnaire examining the extent to which participants agreed with the statement that *“Ramadan seems to come around more quickly each year”*.

In both studies, emotional wellbeing was assessed using the WHO-QOL BREF [40] which gives five sub-measures of quality of life: physical health, psychological health, social relationships, environmental health. This measure was selected instead of individual measures of anxiety or depression as it is correlated with these measures of emotion and provides additional information about social relationships, physical and environmental health which could impact temporal experience. In addition, an event specific measure of emotion was also taken by measuring participants self-reported enjoyment of Christmas/Ramadan. Memory function was assessed using the Prospective Retrospective Memory Questionnaire [41], which provides measures of self-reported prospective and retrospective memory function. Self-reported attention to time was assessed by participants rating the extent to which they thought about the passage of time on a day-to-day basis.

It was expected that there would widespread agreement with the suggestion that Christmas and Ramadan felt like they came around more quickly each year. Based on observations from distortions to short-epochs, it was expected that greater self-reported attention to time and a more positive emotional appraisal of Christmas/Ramadan would be associated with greater agreement that Christmas and Ramadan come around more quickly each year. Conversely, lower quality of life and poorer memory function were expected to be associated with a slowing of the passage of time between annual events.

## Study 1

### Method

#### Participants

One thousand and twenty-two people were recruited through volunteer sampling via posts on Prolific.co and emails to university staff and students. To take part participants had to be aged 18 years or over, currently residing in the UK and with a self-identified religious belief of atheist, Christian or Catholic. Of the initial 1,022 who opened the online questionnaire, 233 participants did not answer every question. These participants were therefore excluded from data analysis. The final sample therefore consisted of 789 participants, 242 (32%) females and 549 (68%) males. The age of participants ranged from 18 to 80 years (*M* = 41.68, *SD* = 16.48). 25% of participants were aged 18–25, 16% were aged 26–35, 14% were aged 36–45, 20% were aged 46–55 and 25% were aged over 55. Recruitment took place between 3^rd^ December 2022 and the 18th of December 2022.

#### Ethics

All participants gave informed consent by ticking an online form before completing the questionnaire. The study was approved by Liverpool John Moores University Research Ethics Committee (22/PSY/068).

#### Measures

Participants completed an online questionnaire distributed through Qualtrics.com. The questionnaire was released to participants on the 3^rd^ December 2022 and closed on the 18th of December 2022. The questionnaire recorded demographic information (age and gender), passage of time judgements, attention to time, enjoyment of Christmas, quality of life and memory function. Quality of life was assessed using the WHO-QOL-BREF [40] and memory function was assessed using the PRMQ [41]. Participants took approximately 8 minutes to complete the questionnaire.

*The passage of time*. The following passage of time judgement (POTJ) question explored participants experience of time and attention to time.

“*To what extent do you agree that Christmas comes around more quickly each year?*”

Participants responded using the following 7 point Likert scale: 1. Strongly disagree, 2. Disagree 3. Somewhat disagree, 4. Neither agree nor disagree, 5. Somewhat agree, 6. Agree, 7. Strongly agree.

“*How much do you think about the passage of time on a day-to-day basis?*”

Participants responded using the following 7 point Likert scale: 1. Never, 2. Rarely 3. Occasionally, 4. Some of the time, 5. Often, 6. Very often, 7. All of the time.

*Enjoyment of Christmas*. The following question explored participants enjoyment of Christmas

“*I really enjoy celebrating Christmas*”

Participants responded using the following 7 point Likert scale: 1. Strongly disagree, 2. Disagree 3. Somewhat disagree, 4. Neither agree nor disagree, 5. Somewhat agree, 6. Agree, 7. Strongly agree.

*Quality of life*. Quality of life was measured using the WHOQOL-BREF [40] which is a 26-item scale derived from the WHO-QOL-100 quality of life scale. The WHOQOL-BREF contains questions assessing overall quality of life and then four subscales assessing satisfaction with 1) physical health (7-items), 2) Psychological health (6-items), 3) Social Relationships (3 items) and 4) Environmental health (8-items).

*Memory function*. Memory function was assessed using the Prospective and Retrospective Memory Questionnaire (PRMQ) [41]. The PRMQ contains 16 items which measure the self-reported frequency of memory slips which can occur during everyday life. Eight items relate to prospective memory failures, for example, “Do you decide to do something in a few minutes’ time and then forget to do it?”. A further 8 items relate to retrospective memory errors, for example, “Do you fail to recognise a place you have visited before?”. Participants respond using a 5-point scale: Very often, quite often, sometimes, rarely, never, resulting in minimum and maximum possible scores of 8 and 40. A higher score therefore equates to greater self-reported memory errors.

#### Data analysis

Non-parametric analyses were used to analyze the data because the main outcome variable, the passage of time, was measured using a single Likert scale. Furthermore, Kolmogorov Smirnov tests confirmed that all outcome variables (POTJ, quality of life, enjoyment of Ramadan/Christmas and attention to time) were not normally distributed (*p* < .001 for all). To assess the relationship between the passage of time and measures of enjoyment, attention to time, quality of life, memory function, Spearman’s correlations were conducted. To assess whether these factors were predictive of the passage of time ordinal logistical regression analyses was conducted.

### Results

**Fig 1:**
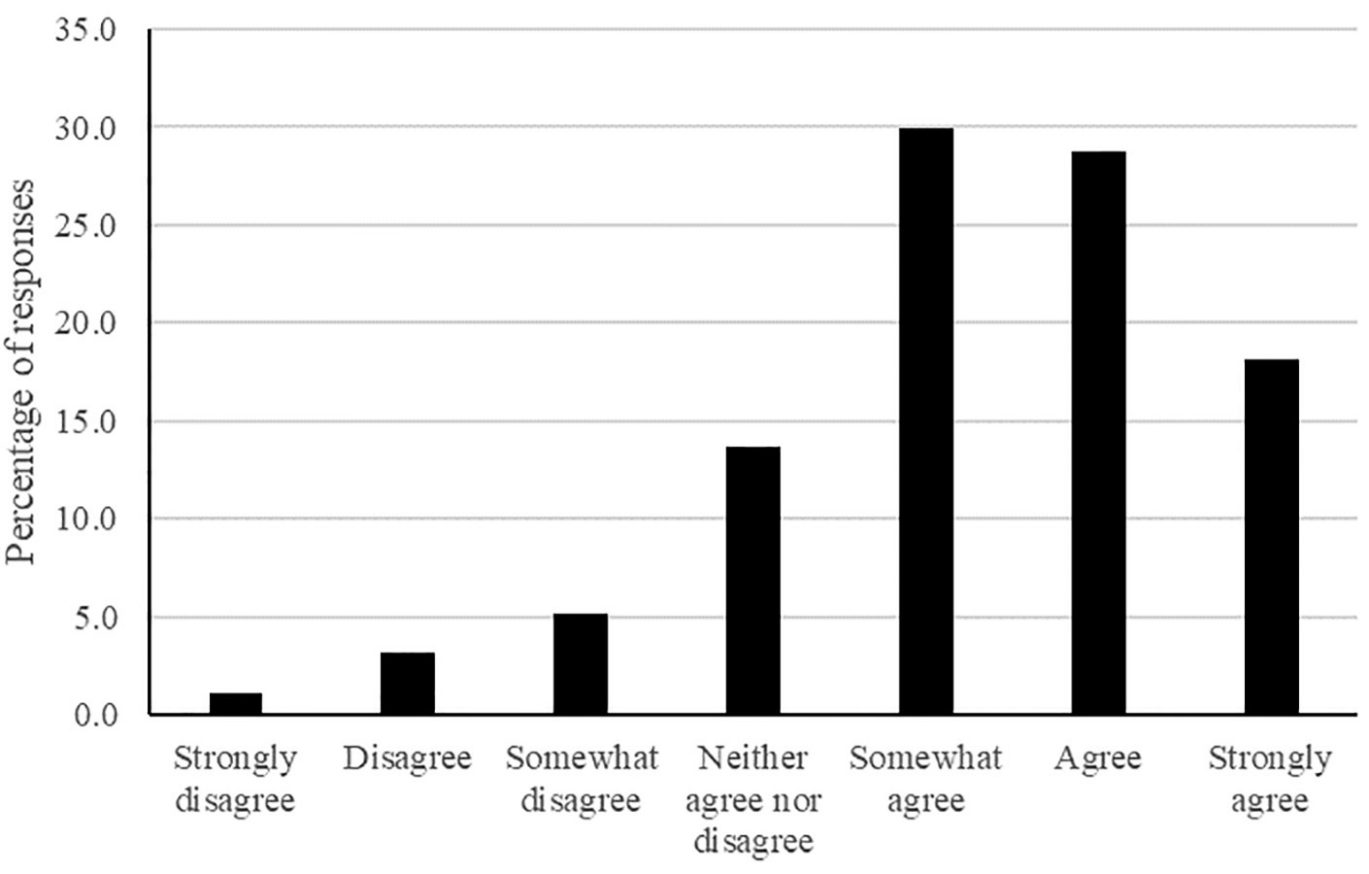
The frequency of responses for each Likert point for the passage of time question for Christmas in the UK.

Examination of Fig 1 suggests that participants from the UK had a strong tendency to believe that Christmas came around more quickly each year. Only 10% of participants disagreed that Christmas comes around more quickly each year, 14% neither agreed nor disagreed, and 76% of participants agreed that Christmas feels like it comes around more quickly each year.

Table 1 shows correlation coefficients for the relationships between POTJ-Christmas and measures memory function, quality of life, age, attention to time and enjoyment of Christmas. There were significant positive relationships between POTJ-Christmas and attention to time and enjoyment of Christmas, prospective memory errors, retrospective memory errors and social quality of life. Greater attention to time on a day-to-day basis, greater enjoyment of Christmas, greater social quality of life and poorer memory function were therefore associated with greater belief that Christmas comes around more quickly each year. There were significant negative relationships between POTJ-Christmas and age and psychological quality of life. Greater belief that Christmas comes around more quickly each year was therefore associated with younger age and lower psychological quality of life.

**Table 1:** Correlation coefficients between POTJ-Christmas, age, memory function, attention and emotional wellbeing.

	Passage of time Christmas	Age	QoL Environment	QoL Social Relationships	QoL Psychological Health	QoL Physical Health	Retrospective Memory	Prospective Memory	Enjoyment of Christmas
Attention to time	.33**	-.10*	-.11**	-.01	-.11*	-.09*	.19**	.17**	.08*
Enjoyment of Christmas	.16**	-.20**	.15**	.27**	.15**	.10*	.02	.08*	
Prospective Memory	.15**	-.34**	-.32**	-.07*	-.41**	-.34**	-.76**		
Retrospective Memory	.11*	-.33**	-.31**	-.12**	-.39**	-.31**			
QoL Physical Health	-.04	.06	.57**	.32**	.58**				
QoL Psychological Health	-.07*	.29**	.64**	.52**					
QoL Social Relationships	.08*	.01	.43**						
QoL Environment	-.03	.20*							
Age	-.10*								

Ordinal regression with proportional odds was conducted to establish the effect of demographic factors, and measures memory function, quality of life, age, attention to time and enjoyment of Christmas on POTJ-Christmas. The model therefore had a dependent variable of the passage of time judgment, and predictor categorical factors of age group (18–25, 26–35, 36–45, 46–55, 55+) and gender (male, female) and covariates of prospective memory score, retrospective memory score, QoL physical health, QoL psychological health, QoL social relationships, QoL environment, attention to time and enjoyment of Christmas.

The model was a statistically significant, χ^2^(10) = 122.59, *p* < .001 fit for the data, with pseudo-R squared values of .05–.15. Table 2 shows that there were four significant predictors of the passage of time; the amount of attention paid to time and the extent to which Christmas was enjoyed, prospective memory function and social QOL. Gender, age, retrospective memory function, and the physical, psychological, and environmental domains of quality of life were not predictive of experiences of the passage of time. Paying greater attention to the passage of time, greater and enjoyment of Christmas, a greater number of self-reported prospective memory errors and higher social QOL were associated with greater belief that Christmas comes around more quickly each year.

**Table 2:** Wald, odds ratios and 95% confidence intervals from the ordinal regressions conducted for the passage of time judgements.

	UK		
Measure	Wald	Odds Ratio	95% CI
Age	.15	1.02	.93–1.12
Attention to time	75.80**	1.58	1.42–1.75
Enjoyment of Christmas	5.02*	1.10	1.01–1.19
Prospective memory	3.97*	1.04	1.00–1.07
Retrospective memory	.25	.99	.96–1.03
QoL Physical	.009	1.00	.99–1.01
QoL Psychological	2.95	.99	.98–1.00
QoL Social	4.45*	1.01	1.00–1.02
QoL Environmental	.36	1.00	.99–1.02
Gender	3.02	.78	.59–1.03

### Discussion

The results of Study 1 suggest that in the UK, there is widespread agreement that Christmas feels like it comes around more quickly each year. Analysis of the factors associated with the belief that Christmas comes around more quickly each year indicated partial support for the hypothesis. As expected, greater enjoyment of Christmas and greater awareness of the passage of time were associated with greater agreement that Christmas comes around more quickly each year. Contrary to expectations, retrospective memory function was not associated with temporal experience, however a greater number of prospective memory errors were associated with the experience of a faster passage of time. The association between better social and relationship health and a faster passage of time confirms previous suggestions that isolation contributes to a slower passage of time. Interestingly, age and the measures of psychological, environmental and physical wellbeing were not related to experiences of time.

## Study 2

The findings of study 1 broadly supported the hypothesis that emotional wellbeing, memory function and attention to time would be predictive of agreement that time distorts resulting in the sensation that Christmas comes around more quickly each year. Study 2 sought to examine whether comparable associations between time experience, attention, memory and wellbeing would be observed for an annual event in a different culture, namely the extent to which people living in Iraq agreed that Ramadan felt like it comes around more quickly each year.

### Method

#### Participants

Six-hundred and twenty-one participants recruited through volunteer sampling via www.sadiq.edu.iq. To take part participants had to be aged 18 years or over, currently residing in the Iraq and with a self-identified religious belief of Muslim. The sample included 208 (33%) females and 413 (67%) males. 22% of participants were aged 25–25, 21% were aged 26–35, 21% were aged 36–45, 19% were aged 46–55 and 17% were aged over 55. Recruitment commenced on the 22nd of January 2023 and ended on the 25th of February 2023.

#### Ethics

All participants gave informed consent by ticking an online form before completing the questionnaire. The study was approved by Liverpool John Moores University Research Ethics Committee (22/PSY/068).

#### Measures

The questionnaire used in Study 1 was translated into Arabic by the research team and references to Christmas were replaced by references to Ramadan. For the WHO-QOL-BREF, the validated Arabic translation was used [42]. For the PRMQ, the Arabic translation provided by Vetrayan et al. [42] was used. Participants completed an online questionnaire distributed through www.sadiq.edu.iq. The questionnaire was released to participants on the 22nd of January 2023 and closed on the 25th of February 2023. Participants took approximately 8 minutes to complete the questionnaire.

#### Data analysis

The data analysis strategy used in Study 1 was replicated in Study 2. To assess the relationship between the passage of time and measures of enjoyment, attention to time, quality of life, memory function, Spearman’s correlations were conducted. To assess whether these factors were predictive of the passage of time ordinal logistical regression analyses was conducted. In addition, Mann-Whitney U tests were used to compare the passage of time, memory function, attention to time, enjoyment and quality of life in the UK and Iraq.

### Results

**Fig 2:**
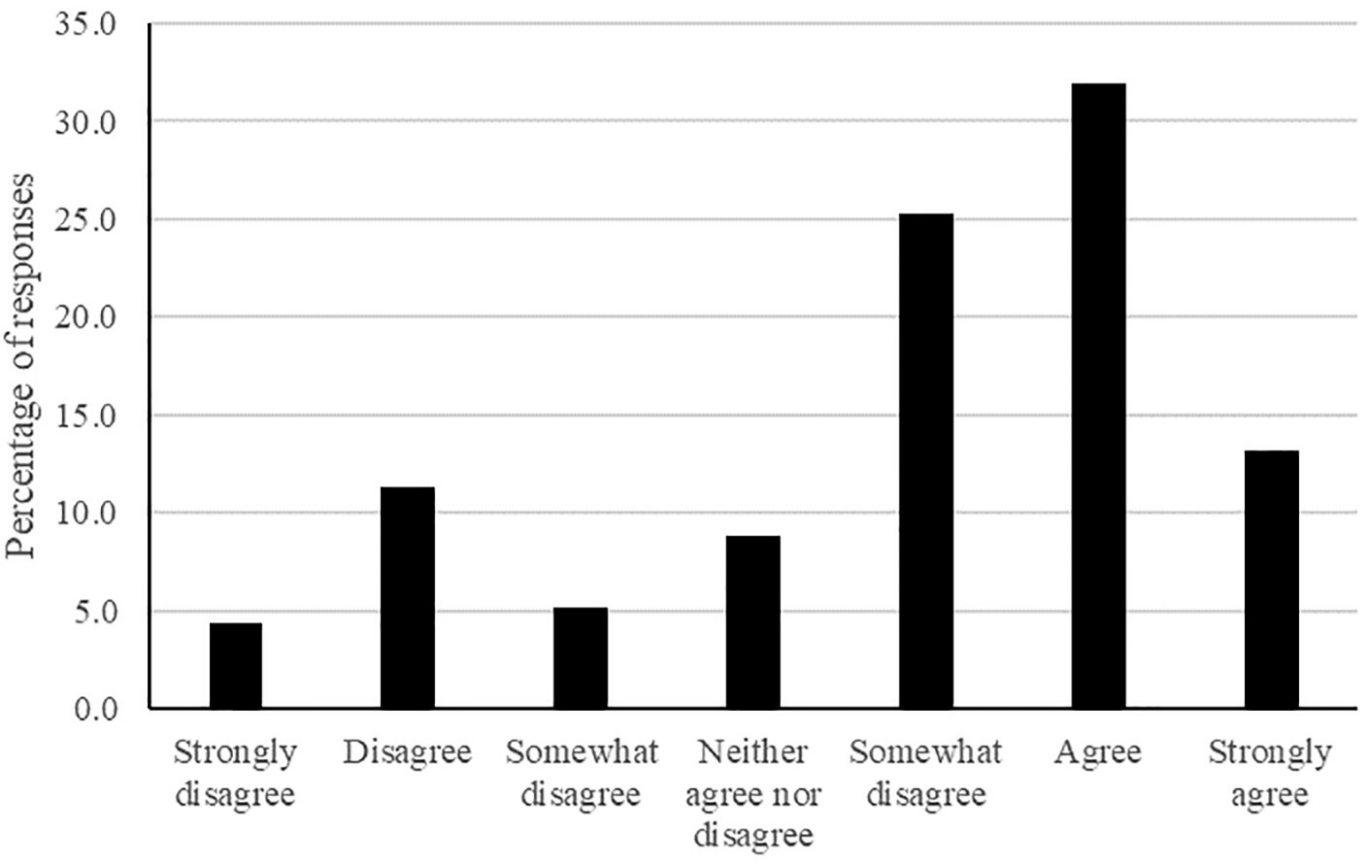
The frequency of responses for each Likert point for the passage of time question for Ramadan.

Examination of Fig 2 suggests that 21% of participants disagreed that Ramadan comes around more quickly each year, 9% neither agreed nor disagreed, and 70% of participants agreed that Ramadan feels like it comes around more quickly each year (Table 3).

**Table 3:** Correlation coefficients between POTJ-Ramadan, age, memory function, attention and emotional wellbeing.

	Passage of time Ramadan	Age	QoL Environment	QoL Social Relationships	QoL Psychological Health	QoL Physical Health	Retrospective Memory	Prospective Memory	Enjoyment of Ramadan
Attention to time	.27**	-.01	-.05	-.04	-.14**	-.14**	.04	.08*	.03
Enjoyment of Ramadan	.08*	-.04	.06	.09*	.15**	.10*	-.06	-.02	
Prospective Memory	.20**	-.14**	-.27**	-.20**	-.35**	-.29**	.83**		
Retrospective Memory	.13**	-.10*	-.27**	-.18**	-.35**	-.24**			
QoL Physical Health	-.10*	.08	.48**	.37**	.48**				
QoL Psychological Health	-.12*	.17**	.57**	.37**					
QoL Social Relationships	-.02	.17**	.39**						
QoL Environment	-.08*	.16**							
Age	-.17**								

Table 4 shows correlation coefficients for the relationships between POTJ-Ramadan and measures memory function, quality of life, age, attention to time and enjoyment of Ramadan. There were significant positive relationships between POTJ-Ramadan and attention to time and enjoyment of Ramadan, prospective and retrospective memory errors. There were significant negative relationships between POTJ-Ramadan and age, and physical, psychological and environmental quality of life. Greater belief that Ramadan comes around more quickly each year was therefore associated great enjoyment of Ramadan, greater attention to time, younger age, greater prospective and retrospective memory errors and reduced physical, psychological and environmental QOL.

**Table 4:** Descriptive statistics for the IRAQ based questionnaire measures.

	Iraq	
Measure	Mean	SD
Passage of time	4.88	1.69
Attention to time	4.07	1.48
Enjoyment of Ramadan	4.44	.77
Quality of life Physical Health	57.82	14.46
Quality of life Psychological Health	55.43	17.15
Quality of life Social Relationships	48.83	19.60
Quality of life Environmental	42.80	16.13
Prospective memory	24.25	6.71
Retrospective Memory	22.53	7.39

Ordinal regression with proportional odds was conducted to establish the effect of demographic factors, and measures memory function, quality of life, age, attention to time and enjoyment of Ramadan on POTJ-Ramadan. The model was a statistically significant, χ^2^(10) = 102.18, *p* < .001 fit for the data, with pseudo-R squared values of .05–.16. Table 5 shows that there were five significant predictors of the passage of time; the amount of attention paid to time and the extent to which Ramadan was enjoyed, prospective memory function, age and gender. Retrospective memory function, and the physical, psychological, social and environmental domains of quality of life were not predictive of experiences of the passage of time. Paying greater attention to the passage of time, greater and enjoyment of Ramadan, and greater self-reported prospective memory errors, younger age and female gender were associated with greater belief that Ramadan comes around more quickly each year.

**Table 5:** Wald, odds ratios and 95% confidence intervals from the ordinal regressions conducted for the passage of time judgements.

	Iraq		
Measure	Wald	Odds Ratio	95% CI
Age	11.33**	.98	.97–.99
Attention to time	37.18**	1.37	1.24–1.51
Enjoyment of Ramadan	9.45**	1.33	1.11–1.60
Prospective memory	12.35**	1.07	1.03–1.11
Retrospective memory	1.80	.98	.94–1.01
QoL Physical	.24	1.00	.99–1.01
QoL Psychological	.08	1.00	.99–1.01
QoL Social	1.34	1.00	.99–1.01
QoL Environmental	.15	1.00	.99–1.01
Gender	5.16*	1.44	1.05–1.97

#### Comparison of Study 1 and Study 2

Table 6 shows comparisons between scored from the UK and Iraq for the passage of time, quality of life, memory function, enjoyment and attention to time. Examination of Table 6 reveals a number of key differences between the samples. A Mann Whitney U test compared the passage of time in the UK and Iraq (*U =* 221992.60, *p* = .002). The UK sample reported significantly greater agreement that Christmas feels like it comes around more quickly each year than the Iraqi population reported for Ramadan. Further Mann Whitney U tests were used to compare quality of life and memory function in the UK and Iraqi samples. Physical (*U =* 14855.50, *p* < .001), social (*U =* 154380.50, *p* < .001) and environmental (*U =* 81986.00, *p* < .001) quality of life were significantly greater in the UK than Iraq. There was no significant difference in psychological quality of life between the two countries (*U =* 239762.00, *p* = .52). PRMQ scores were significantly greater in the UK than Iraq, suggesting significantly worse memory function in the UK population for prospective (*U =* 199667.50, *p* < .001) and retrospective (*U =* 167234.00, *p* < .001) memory function. Mann-Whitney U tests were used`to compare attention to time and enjoyment of Christmas/Ramadan in the UK and Iraq. Attention to time did not differ significantly across the two countries (*U =* 242159.50, *p* = .73). Enjoyment of Christmas was however reported as significant greater in the UK than enjoyment of Ramadan in Iraq (*U =* 145644.50, *p* < .001).

**Table 6:** Descriptive statistics for the UK based questionnaire measures.

	UK	
Measure	Mean	SD
Passage of time	5.27	1.32
Attention to time	4.04	1.32
Enjoyment of Christmas/Ramadan	5.21	1.66
Quality of life Physical Health	68.58	17.64
Quality of life Psychological Health	54.60	18.26
Quality of life Social Relationships	62.07	20.37
Quality of life Environmental	64.07	14.79
Prospective memory	21.98	6.19
Retrospective Memory	18.56	5.61

Correlates and predictors of the passage of time

### Discussion

The results of Study 2 suggest that there was widespread agreement that Ramadan feels like it comes around more quickly each year. As expected, greater enjoyment of Ramadan, greater attention to time, female gender, greater numbers of self-reported prospective memory errors and reduced age were all associated with greater belief that Ramadan comes around more quickly each year. The findings broadly support existing literature suggesting that increased attention to time, emotion and memory function can contribute to distortions to time. Interestingly, decreased age was associated with greater agreement that Ramadan comes around more quickly. Contrary to previous literature suggesting that time speeds up with increasing age, the current findings suggest greater disagreement with time speeding up with increasing age.

## General discussion

This study sought to explore distortions to the passage of time for annual events. Specifically, it aimed to establish whether factors such as age, memory function, quality of life, enjoyment and attention to time were associated with the subjective sensation that annual events appear to come around more quickly each year.

The results show that there was widespread agreement that annual events appeared to come around more quickly each year. In the UK, 76% of participants agreed that Christmas appears to come around more quickly each year, in Iraq, 70% of participants agreed that Ramadan appears to come around more quickly each year. The sensation is not therefore limited to a single culture or annual event.

In the UK, the sensation that Christmas comes around more quickly each year was associated with greater attention to time, greater enjoyment of Christmas, a greater number of self-reported prospective memory errors, and greater social quality of life. Age, gender, self-reported retrospective memory errors, and physical, psychological and environmental wellbeing were not predictive of agreement that Christmas appears to come around more quickly each year. In Iraq, the sensation that Ramadan comes around more quickly each year was associated with reduced age, greater attention to time, greater enjoyment of Ramadan, a greater number of self-reported prospective memory errors, and female gender. Self-reported retrospective memory errors and quality of life were not predictive of agreement that Ramadan appears to come around more quickly each year. Together, these findings show that despite the sensation that annual events come around more quickly each year being anecdotal, it is predicted by established psychological constructs. This suggests that anecdotes such as these appear to be grounded in individual psychological experiences rather than being simple adages with little or no psychological correlates.

The factors which predicted the passage of time for Christmas and Ramadan partially supported the hypothesis. The association between greater enjoyment of Christmas/Ramadan and greater agreement that the events come around more quickly each year supports the hypothesis that positive emotion would be associated with a faster passage of time. The findings are therefore consistent with previous studies demonstrating that positive emotion is associated with a faster passage of time [1–11, 18, 19]. The absence of consistent relationships between quality of life and the passage of time suggests that event specific emotional arousal rather than more generic measures of wellbeing are determinant of the passage of time between annual events.

The positive association between attention to time and agreement that annual events are coming around more quickly suggests that greater attention to time does not always result in a sensation of time slowing down. Boredom and clock-watching, both of which are associated with increased attention to time, have both been shown to result in a slowing of the passage of time [43–45]. Findings such as these have contributed suggestions that there is a negative relationship between the speed of the passage of time and attention to time. The findings from the current study however show that, when considering the passage of time to a future event, greater attention to time is associated with a faster passage of time. This is consistent with the effects of time pressure on time experience in which greater time pressure is associated with greater attention to time and a sensation that time is passing quickly [11, 35–39].

The absence of an association between self-reported retrospective memory errors and the passage of time suggests that the role of memory function in experiences of time may be more complex than previously imagined. Memory storage and contextual change models of the passage of time suggest that the amount and type of information stored in memory is associated with the sensation of “more time” having occurred. These theories imply that retrospective memory function should therefore be an important factor for temporal experience. In the current study however, prospective rather than retrospective memory function was associated with the passage of time. In the current study, the role of prospective rather than retrospective memory function in temporal experience may reflect the future focused nature of the passage of time question posed.

Much of the existing research into the factors which affect the passage of time has asked participants to make a retrospective passage of time judgment i.e. how quickly has today passed in comparison to normal, or, a present passage of time judgment i.e. how quickly is time passing now in comparison to normal [1–10]. The current study however asked participants to judge the passage of time for a future event, i.e. do the up-coming Christmas/Ramadan feel like they are coming around more quickly than normal. Whilst answering this question may require people to use retrospective memory to recall information about past Christmases/Ramadan’s, it also likely requires participants to project to the upcoming event. The future focus may therefore explain why prospective rather than retrospective memory function was determinant of the passage of time.

The association between prospective memory function, greater attention to time and a faster subjective passage of time therefore perhaps reflects increasing time pressure in the run up to Christmas and Ramadan. Data from the current studies was collected two months before Christmas day and the start of Ramadan. At this point, the significant preparations required for the events would be taking place and these likely placed significant prospective memory burden on participants.

Interestingly, the results show that older age was not associated with greater belief that Christmas or Ramadan come around more quickly each year. Instead, in Iraq, younger age was associated with greater agreement that Ramadan comes around more quickly each year and old age was associated with greater disagreement with this. In the UK, age was not predictive of beliefs about the passage of time for Christmas. These findings add to a growing body of literature which either fails to show an effect of age on the passage of time [2, 18], or reveals a negative association between age and the passage of time i.e. that time slows down as age increases [1, 17]. Further systemic assessment of how and when age alters the passage of time is therefore warranted.

Whilst this study demonstrates that experiences of time for annual events appears to be influenced by memory function, attention and event specific enjoyment, it is possible that the formulation of the question reflects participants agreement with a stereotype (i.e. the belief that Christmas/Ramadan come around more quickly each year) rather than their actual lived experience of time passing more quickly since the previous Christmas/Ramadan. Future research should therefore seek to establish the extent to which lived experiences of time correspond to agreements with stereotypes about time.

### Limitations

This study compared beliefs about the passage of time for Ramadan and Christmas. Whilst these are both large significant events, there are notable differences between the two. Firstly, the precise timing of Ramadan is based on a lunar cycle and therefore varies from year to year, with the precise date of the start and end often not known until very short notice before. The lunar cycle also means that the timing of Ramadan changes across a lifespan and is not therefore associated with a specific month or even season. Christmas however, occurs on a fixed date each year. There is therefore a greater degree of temporal change and uncertainty for Ramadan than there is for Christmas, and this may influence the way in which time is experienced in anticipation of Ramadan and in between Ramadan’s. In the UK Christmas consists of three core days of activity; Christmas eve, Christmas day and Boxing day. These days are often indulgent, involving socialisation and feasting. Ramadan however is a month-long event requiring significant sacrifice in the form of daily fasting. The differences in the length of Christmas and Ramadan, and the activities performed during each period mean that the two events are not directly comparable. It is therefore possible that event-based differences contributed to differing predictors of the passage of time in the two countries.

In the current study, the passage of time was assessed using a single Likert scale. Whilst this is consistent with other studies exploring the passage of time during everyday life [1–9, 18, 19, 24, 25] it is possible that the uni-dimensional nature of this assessment failed to completely capture all aspects of participants experiences of the passage of time. Future research should therefore seek to develop and validate multi-item measures to assess subjective experiences of the passage of time.

Although the regression models were significant predictors of experiences of the passage of time, the odds ratios produced in each model were small. This suggests that other variables, not explored in the current study, may be determinant of the passage of time for annual events. Further research should therefore seek to establish how a wider range of factors influence experiences of the passage of time. This should be achieved through a combination of cross-sectional analysis of a broader range of predictor factors, and laboratory-based work manipulating and measuring the impact of factors known to alter temporal experience, such as physiological arousal [19] and cognitive load [34].

The widespread agreement that Christmas and Ramadan felt like they were coming around more quickly each year may have been enhanced by growing trends for marketing campaigns for annual events such as Christmas, Ramadan, Halloween and Easter to start earlier and earlier each year [46]. Nowadays, in the UK for example, it is not uncommon to see Christmas products in stores in September, three months before Christmas itself. The belief that Christmas and Ramadan come around more quickly each year may not therefore just reflect a subjective experience, but may also reflect the reality of changing marketing practices. However, even if these changes contribute to the general sensation that Christmas and Ramadan come around more quickly each year, they do not explain why this belief is greater in people with poorer memory function and greater attention to time, as both of these factors are not themselves related to Christmas and Ramadan. Instead, it would appear that there is a psychological basis which determines the extent of the belief.

## Conclusions

The results of this study suggests that there is widespread belief that annual events such as Christmas and Ramadan feel as though they come around more quickly each year. The sensation is not therefore limited to a single culture or event. The extent to which people agreed that these events appear to occur more quickly each year was predicted by prospective memory function, event specific enjoyment and attention to time. Critically, older age was not associated with greater speeding up of time between events in either country. These findings highlight that our experience of time is not just influenced by what we have already done, but also our capacity to remember what remains to be done. Further research exploring the role of prospective memory in timing is therefore warranted.

## Supporting information

S1 Data Supporting data. (SAV)
